# HAHNet: a convolutional neural network for HER2 status classification of breast cancer

**DOI:** 10.1186/s12859-023-05474-y

**Published:** 2023-09-20

**Authors:** Jiahao Wang, Xiaodong Zhu, Kai Chen, Lei Hao, Yuanning Liu

**Affiliations:** 1 College of Software, Jilin University, Changchun, 130012 China; 2 Key Laboratory of Symbolic Computation and Knowledge Engineering of Ministry of Education, Jilin University, Changchun, 130012 China; 3 College of Computer Science and Technology, Jilin University, Changchun, 130012 China

**Keywords:** Breast cancer, HER2, Deep learning, HAHNet

## Abstract

**Objective:**

Breast cancer is a significant health issue for women, and human epidermal growth factor receptor-2 (HER2) plays a crucial role as a vital prognostic and predictive factor. The HER2 status is essential for formulating effective treatment plans for breast cancer. However, the assessment of HER2 status using immunohistochemistry (IHC) is time-consuming and costly. Existing computational methods for evaluating HER2 status have limitations and lack sufficient accuracy. Therefore, there is an urgent need for an improved computational method to better assess HER2 status, which holds significant importance in saving lives and alleviating the burden on pathologists.

**Results:**

This paper analyzes the characteristics of histological images of breast cancer and proposes a neural network model named HAHNet that combines multi-scale features with attention mechanisms for HER2 status classification. HAHNet directly classifies the HER2 status from hematoxylin and eosin (H&E) stained histological images, reducing additional costs. It achieves superior performance compared to other computational methods.

**Conclusions:**

According to our experimental results, the proposed HAHNet achieved high performance in classifying the HER2 status of breast cancer using only H&E stained samples. It can be applied in case classification, benefiting the work of pathologists and potentially helping more breast cancer patients.

## Introduction

Breast cancer is the most common cancer in women [1–4] and represents one of the leading causes of death in women [5]. In 2020, breast cancer occupied 12% of all human malignant tumor cases [6], and by 2040, this number is expected to rise to 46%. Human epidermal growth factor receptor-2 (HER2) is a diagnostic and prognostic factor for breast cancer, and HER2-positive breast cancer is one of the several subtypes of breast cancer, which accounts for about 15% of early-stage breast cancers [7]. HER2-positive breast cancer is defined as HER2 gene amplification or HER2 protein overexpression, and HER2-positive tumors grow faster and spread more easily than HER2-negative tumors [8], but the good news is that these tumors can respond better to targeted drugs [9]. Trastuzumab, a HER2-targeted drug, has recently been introduced and greatly improved the survival of HER2-positive breast cancer patients [10]. The treatment plans for breast cancer patients should be formulated based on HER2 status, in this regard, the early diagnosis of HER2 status is crucial, which can greatly improve patient survival.

In the routine diagnosis of HER2 expression, breast cancer tissue sections are stained with hematoxylin and eosin (H&E), the morphology is determined by manual visual inspection, and the expression levels of HER2-specific proteins are then measured by immunohistochemical (IHC) analysis and in situ hybridization (ISH) technology [11]. HER2 expression levels are categorized by the American Society of Clinical Oncology/College of American Pathologists (CAP/ASCO) into four categories (0, 1+, 2+, 3+) based on visual analysis of IHC histological images [11], where patients with expression levels of 0 and 1+ are defined as HER2-negative (HER2−), and those with an expression level of 3+ are classified as HER2-positive (HER2+). Due to the unclear expression of HER2-specific proteins in 2+ cases, further evaluation of HER2 gene status using ISH is required. However, IHC is associated with high costs, besides, the tissue availability, as well as operation skills and analysis of the operator in manual testing can also affect the assessment of HER2 status [14,18], which can have an impact on the final treatment plan.

Deep learning (DL) is under rapid development in recent years and plays a role in various fields. Convolutional Neural Network (CNN) is a DL network model, which is extensively proved to be applicable in multiple research directions such as cell segmentation, tumor classification, and cancer localization. CNN can identify histopathological abnormalities in routine H&E images related to the presence of atomic biomarkers in a range of cancer types, including rectal cancer [12], lung cancer [13], prostate cancer [14], and skin cancer [15]. DL has also been applied in the direction of breast cancer tissue histopathology identification [16]. These works can help reduce the burden on pathologists, and meet the requirements of high precision and efficient computation.

Some solutions to these problems have been proposed. For instance, Kather et al. [17] proposed a deep-learning approach for the assessment of hormone receptor status from H&E stained whole slide images (WSIs). Oliveira et al. [18] developed a CNN model based on multi-instance learning to classify HER2− or HER2+ from H&E images, and their trained model was tested on the CIA-TCGA-BRCA (BRCA) dataset, yielding an accuracy of 83.3%. However, they only classified HER2− and HER2+ but not classified the four statuses of HER2. In [19], U-Net was utilized to find the location of cell nuclei in H&E stained images, and a cascaded CNN architecture was constructed to classify HER2, which resulted in an area under the curve (AUC) value of 0.82 in the Warwick dataset [20]. Nonetheless, in this method, it is necessary to analyze the prediction results of patch-level images during the classification process, which increases the overheads. Moreover, Sakib Hossain Shovon et al. [21] put forward an improved TL architecture HE-HER2Net, using the same BCI dataset as ours, and the accuracy rate reached 87%, this work has achieved promising results, but the prediction accuracy still needs to be improved. Compared with HAHNet proposed in this paper, HE-HER2Net had lower accuracy. Lu et al. [19] proposed a GNN model-SlideGraPh+, which not only predicted the DAB density of H&E stained WSIs, but also predicted the HER2 status according to the DAB density. Thereafter, the trained model was tested on HER2C and Nott-HER2 datasets, yielding the AUCs of 0.78 and 0.8. But the HER2 scores of 2+ cases were avoided during the model testing process. Shamai [22] raised a deep CNN model based on residual networks (ResNet) [23], aiming to predict the expression of molecular organisms in breast cancer by the analysis of digitized H&E stained tissues, where the AUC for HER2 status classification was 0.74. Nevertheless, this work was based on a single data source and only included tissue microarrays (TMA) images.

Based on the above conclusions, existing methods for HER2 status classification of H&E stained images have several limitations, including insufficient granularity in classification, higher computational costs due to patch-level predictions, avoidance of certain class data, and inadequate accuracy. Considering the significance of HER2 status diagnosis and the limitations of current computational methods, we propose the neural network model HAHNet to aid pathologists in better assessing HER2 status and to help more breast cancer patients receive improved treatment plans. In summary, the main contributions of this paper can be summarized as follows.

- A deep learning method is proposed based on attention mechanism and multi-scale feature fusion, so as to improve the accuracy of breast cancer HER2 status classification.
- Unlike most current algorithms, the data predicted by HAHNet are based on conventional H&E images without IHC staining, which increases the difficulty in our prediction, but HAHNet still performs efficiently.

## Materials and methods

### Datasets and pre-processing

**Fig. 1:**
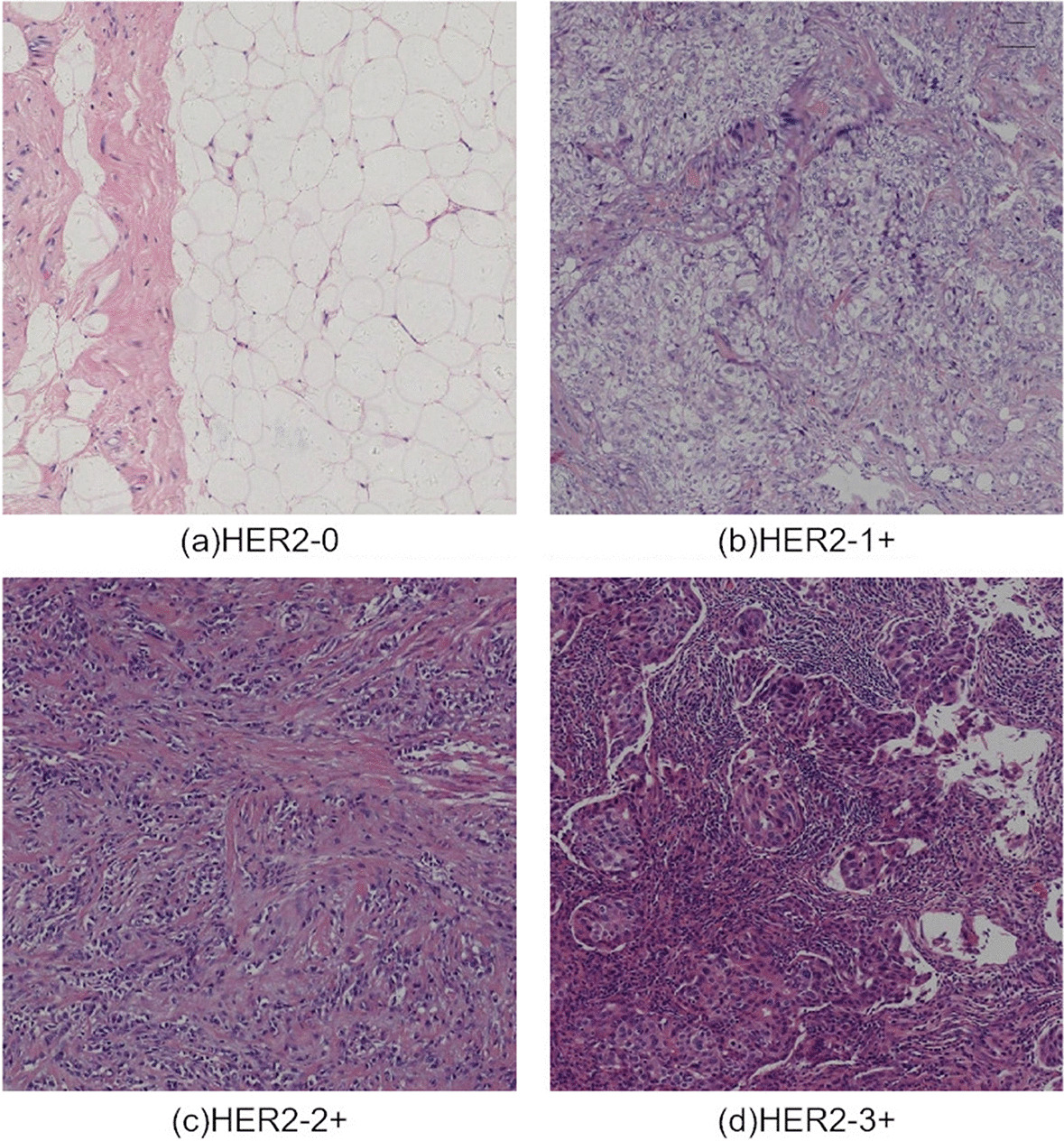
Four types of HER2 image samples in BCI-H&E dataset: **a** HER2-0, **b** HER2-1+, **c** HER2-2+, **d** HER2-3+

The dataset used in our study was a new breast cancer immunohistochemistry (BCI) benchmark dataset [24], and Hamamatsu NanoZommer S60 was utilized to acquire this dataset, with a scan speed of 60 s per slice and a scan resolution of 0.46 um per pixel. This dataset collated 4870 pairs of H&E and IHC images (resolution, 1024*1024). The four types of HER2 H&E images are displayed in Fig. 1.

**Fig. 2:**
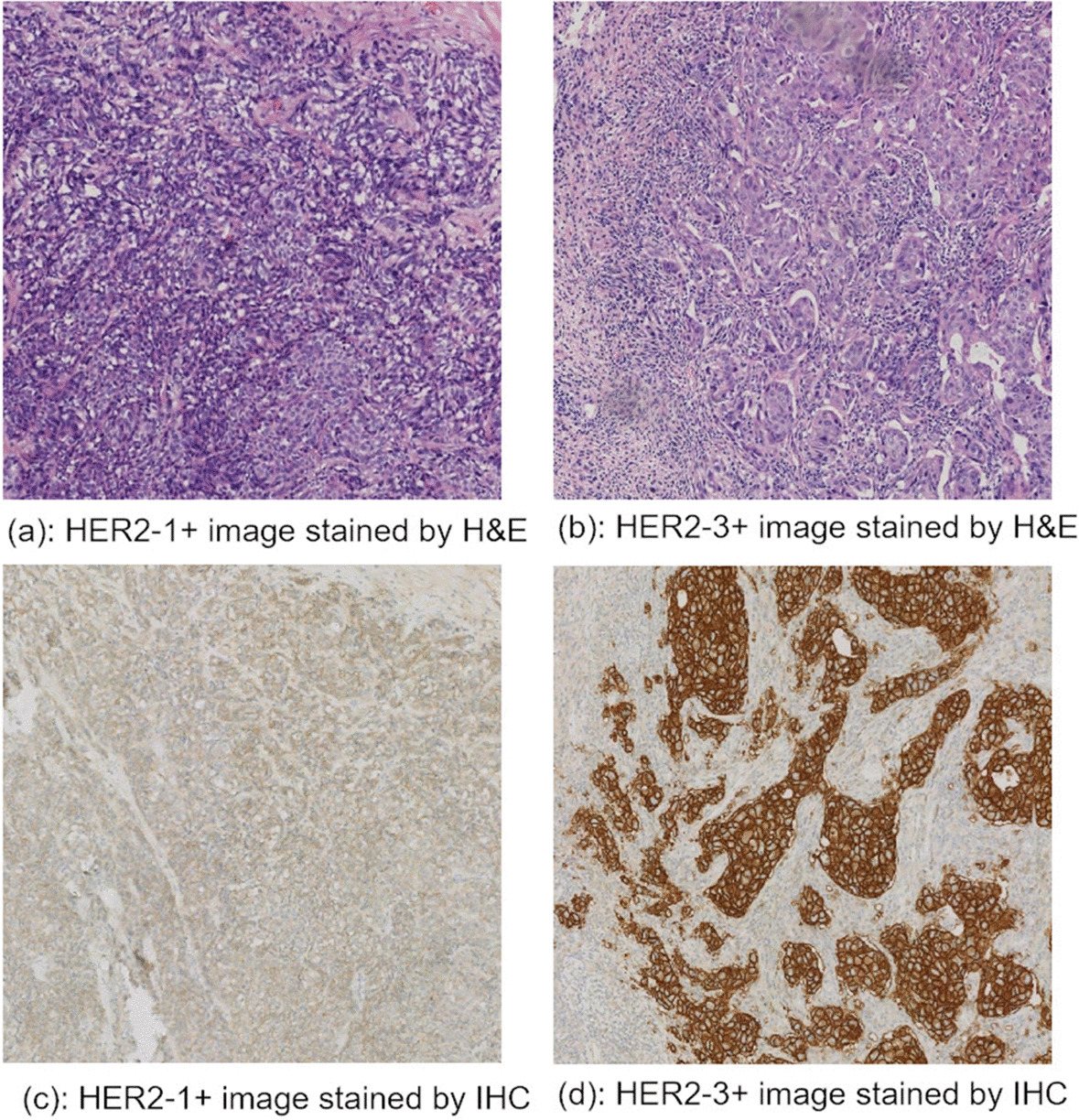
H&E staining images and IHC staining images, **c** and **d** are obtained from **a** and **b** after IHC staining

H&E images from the BCI dataset were adopted in this work. There were 4870 images in total, including 3896 images in the training set and 977 in the test set. To train more efficiently and speed up data processing, the Python toolkit opencv was employed to reduce the size of the data from 1024*1024 to 299*299. Figure 2 presents the H&E images of HER2-1+ and HER2-3+ categories and their images after IHC staining. (a) and (b) displayed the ordinary H&E images, the HER2 status in (a) was 1+, and the HER2 status in (b) was 3+, while (c) and (d) were derived from (a) and (b) after IHC staining. Obviously, (c) and (d) were very different from each other, and the HER2 status in these two images was distinguishable by the naked eye, but it was difficult to distinguish the HER2 status in H&E images without IHC staining.

### HAHNet

**Fig. 3:**
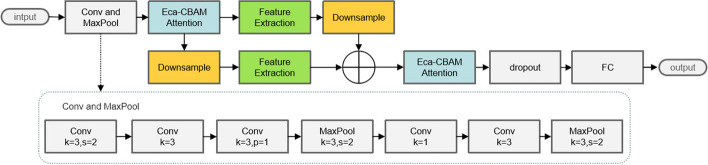
The overall structure of the model. The model takes input images of size $299 \times 299 \times 3$ and outputs four categories corresponding to the four levels of HER2 expression. The Conv and MaxPool module consists of five convolutional layers and two max pooling layers. The FC module consists of only one fully connected layer

As discovered after comparison, the accuracy of InceptionV3 [25] in the evaluation of HER2 status was significantly superior to other classical models. The InceptionV3 model is the third-generation model in the Google Inception series. Compared with other neural network models, the most significant feature of the Inception network is that it expands the convolution operation between neural network layers and realizes multi-scale feature extraction. The idea of multi-scale feature extraction can not only be used in the construction of convolutional modules, but also be applied in the construction of the overall network model structure, as did in this study. HAHNet changed the original model structure of InceptionV3 into a parallel structure, and after convolutional preprocessing, the feature maps were downsampled to extract multi-scale features, which allowed us to obtain more multi-scale features and ensured the accuracy of HAHNet for the classification of HER2 status. The “Inception” structure was adopted in all of our convolution modules. It used the parallel convolution with different-size convolution kernels, and a maximum pooling layer was added to the parallel line for multi-scale feature extraction on the feature map. We also introduced a novel attention mechanism in our model, which combines the Convolutional block attention module (CBAM) [26] and Efficient Channel Attention (ECA) [27]. We named it the Efficient Channel Attention-Convolutional block attention module (ECA-CBAM) attention mechanism. The overall model structure is exhibited in Fig. 3.

#### Feature extraction

Feature Extraction was the most important part of the model. A parallel structure was adopted in all the five modules in Feature Extraction.

**Fig. 4:**
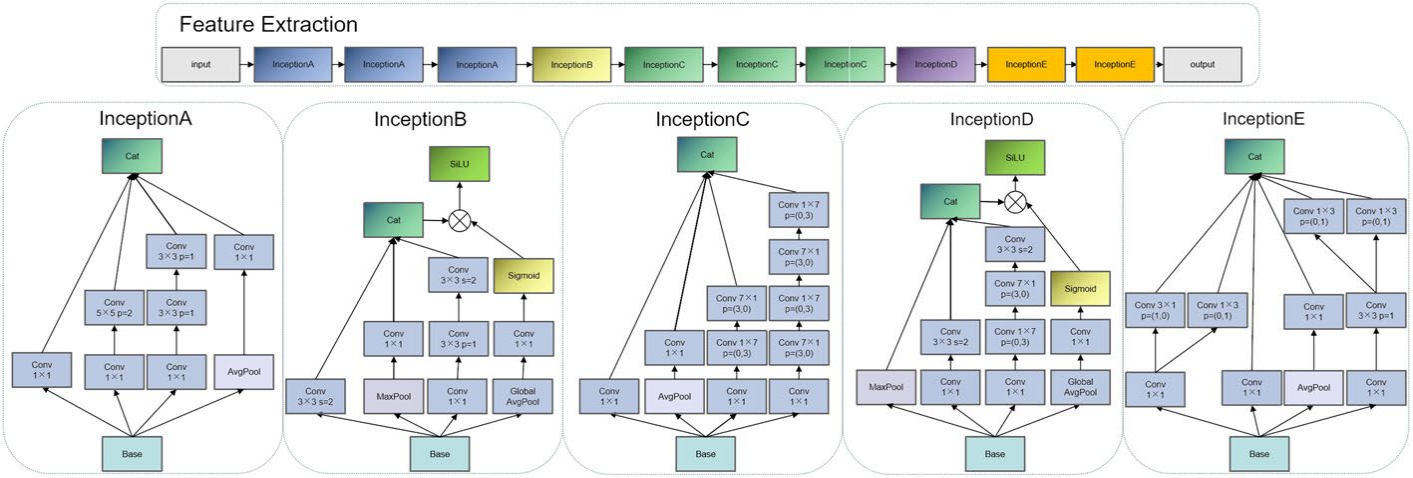
Feature extraction structure, the feature extraction phase consists of five types of convolutional modules: InceptionA, InceptionB, InceptionC, InceptionD, and InceptionE. All of these convolutional modules employ a parallel structure

To be specific, InceptionA, InceptionC, and InceptionE utilized convolutions of different sizes to extract multi-scale features in the four feature extraction lines, introduced an average pooling layer to compress the features, finally fused the results of the four lines and input them into the next module. InceptionB and InceptionD, which included downsampling operations, were the modules that we paid more attention to. Both InceptionB and InceptionD contained four feature extraction lines. Of them, the first three lines adopted convolutions of different sizes to extract multi-scale features and introduced a max pooling layer to extract texture information. To further preserve global information in the dimensionality reduction process, the global average pooling, a 1×1 convolution, and a Sigmoid function were introduced in the fourth line. Notably, the global average pooling contributes to effectively extracting global spatial information. Thereafter, multi-scale features extracted by the first three lines were fused. The results processed by the fourth line were not directly connected with the results of other layers, instead, they were multiplied with the connection results of other layers to adjust the global spatial weight. Later, the final results were incorporated into the SiLU function. The specific structure is shown in Fig. 4.

#### DownsampleBlock

**Fig. 5:**
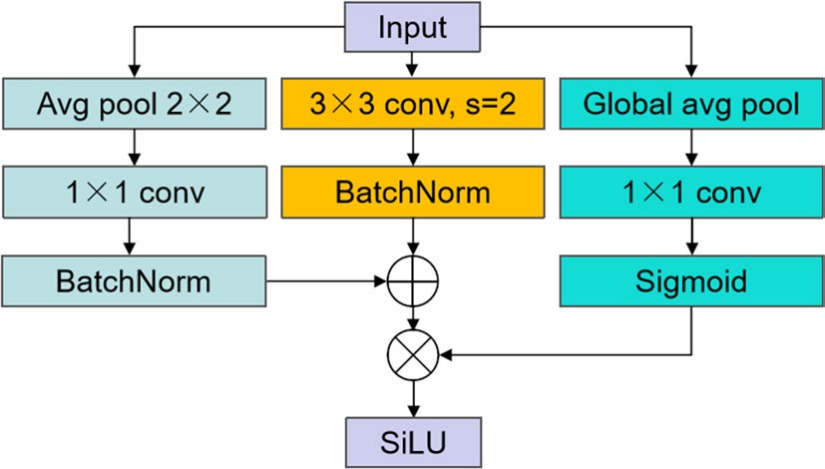
DownsampleBlock structure

Noteworthily, considering that some fine features might be lost in ordinary convolutional downsampling during resolution degradation, Ankit Goyal et al. [28] proposed an ’Inception’ structured downsampling block that implemented the multiscale processing of the feature map during downsampling, where convolutional nuclei with smaller size were able to better sense fine features and preserve them. This block was used in our model. The first line of the downsampling block consisted of a 2D average pooling and a 1×1 convolution, while the second line included a 3×3 convolution with a stride of 2, and the third line contained the global Average pooling, a 1×1 convolution and a sigmoid function. After finishing convolution of the first two lines, BatchNorm was added. Afterwards, the results of the first two lines were fused and multiplied with those of the third line, and the results were later input into the SiLu activation function. Multi-dimensional convolution can help the model to better retain detailed features and context information during the downsampling process in the meantime of adding more activation functions to introduce stronger nonlinear characteristics, which can effectively improve the model learning ability. The specific structure of DownsampleBlock is displayed in Fig. 5.

#### Eca-CBAM attention block

**Fig. 6:**
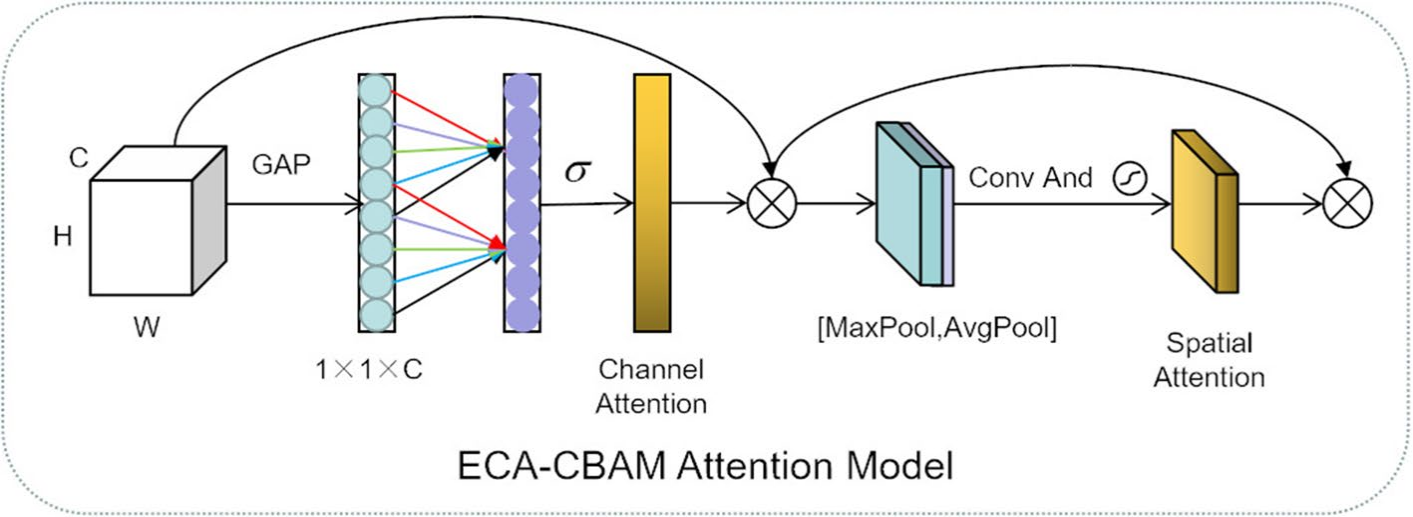
Eca-CBAM attention mechanism structure

Before downsampling, an improved attention block was added, which allowed our model to focus more on important features and better retain important features during downsampling. Based on the purpose of the research, the introduction of spatial attention and channel attention was the best choice, which preserved contextual information and allowed the model to know which features were more important. The CBAM attention mechanism [26] combines spatial attention with channel attention, which meets our needs, but the channel attention in CBAM will perform dimensionality reduction operations, which will bring side effects. Such operations will lead to the loss of some detailed features. Therefore, some improvements to CBAM were made by introducing ECANet [27] channel attention to replace the channel attention in CBAM. The ECA (Efficient Channel Attention) mechanism utilizes 1D convolution to achieve local cross-channel interaction and extract dependencies between channels. This method effectively solves the issues caused by dimension reduction operations. The improved attention mechanism was called Eca-CBAM. The structure of Eca-CBAM attention mechanism is shown in Fig. 6. Channel attention and spatial attention were calculated as follows (Eqs. 1–2).

1 $$\begin{aligned}&\omega =\sigma \left( C1D{k}\left( y \right) \right) \end{aligned}$$

Equation 1 is the calculation formula of channel attention, where C1D denotes 1D convolution, $\sigma$ represents the sigmoid function, and k indicates that the module only involves k parameters, and y represents the input feature map. C1Dk(y) denotes mapping the input feature map y to a vector of dimension k. The Channel Attention Layer is a component of the Eca-CBAM Attention Block, which helps the model focus on relevant channels and suppress less important channels.

2 $$\begin{aligned}&{\text {Ms}}(F)=\sigma \left( f^{7 * 7}([{\text {AvgPool}}(F), {\text {MaxPool}}(F)])\right) =\sigma \left( f^{7 * 7}\left( \left[ F_{\text{ avg } }^{S} ; F_{\max }^{S}\right] \right) \right) \end{aligned}$$

Equation 2 represents the calculation formula of spatial attention, where ? stands for the sigmoid function, *AvgPool* represents average pooling operation, *MaxPool* represents maximum pooling operation, and $f^{7 * 7}$ represents a convolution operation with a filter size of $7 \times 7$. The Spatial Attention Layer is a component of the Eca-CBAM attention block, used to capture spatial dependencies in the input feature map, enabling the model to focus on relevant spatial regions.

## Results

### Experimental setup and parameter initialization

**Fig. 7:**
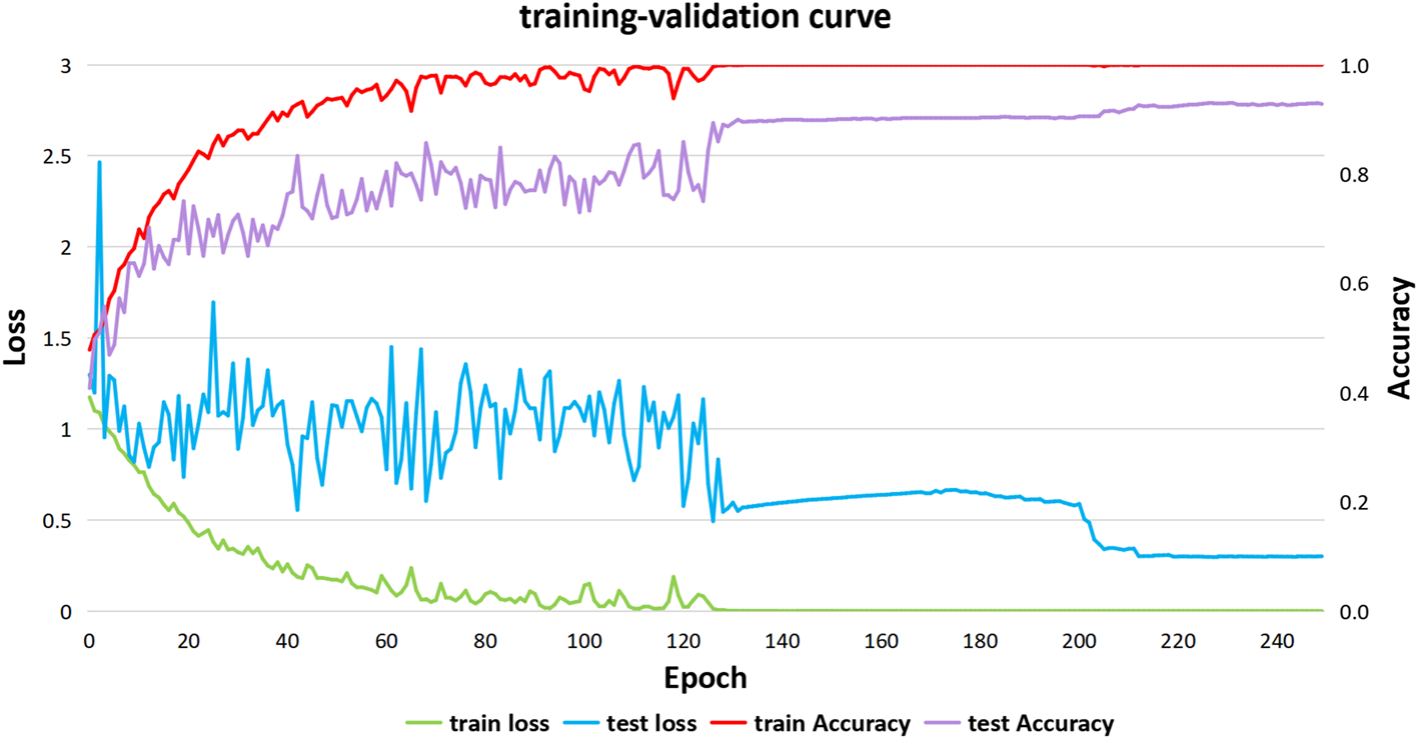
Training-validation curve of HAHNet. This figure illustrates the changes in the train loss, test loss, train accuracy, and test accuracy of HAHNet as the number of epochs increases during the training process

The GPU used in the experiment is RTX4090, the graphics card memory is 24 GB, and the PyTorch deep learning library is utilized in all experiments. The same optimizer (Adam) is used in all models during training. After debugging different hyperparameters, the learning rate of 0.0005. In the loss function, weight decay is a coefficient placed before the regularization term. Its role is to adjust the impact of model complexity on the loss function and prevent overfitting. We set the weight decay to 0.00001. In addition, to prevent overfitting, we added a dropout layer before the fully connected layer. Due to the low learning rate, the model converges slowly, so we set a larger epoch value to ensure full convergence of the model. The epoch value is set to 250. During training, the batch size is set to 64. The input image size is $299 \times 299 \times 3$. In the experiment, the whole slide is used for training and testing without partitioning it into patches. At last, the model weight corresponding to the iteration with the highest precision is retained. To detect the presence of overfitting, we recorded the accuracy and loss rates during the model training process. Figure 7 represents the training-validation curve, displaying accuracy and loss rates during the experiment. It can be observed that the curves initially exhibit fluctuations but eventually smooth out, indicating that the model converges effectively without overfitting or underfitting issues. The highest model accuracy and lowest training loss were achieved at epoch 233.

### Performance metrics

**Fig. 8:**
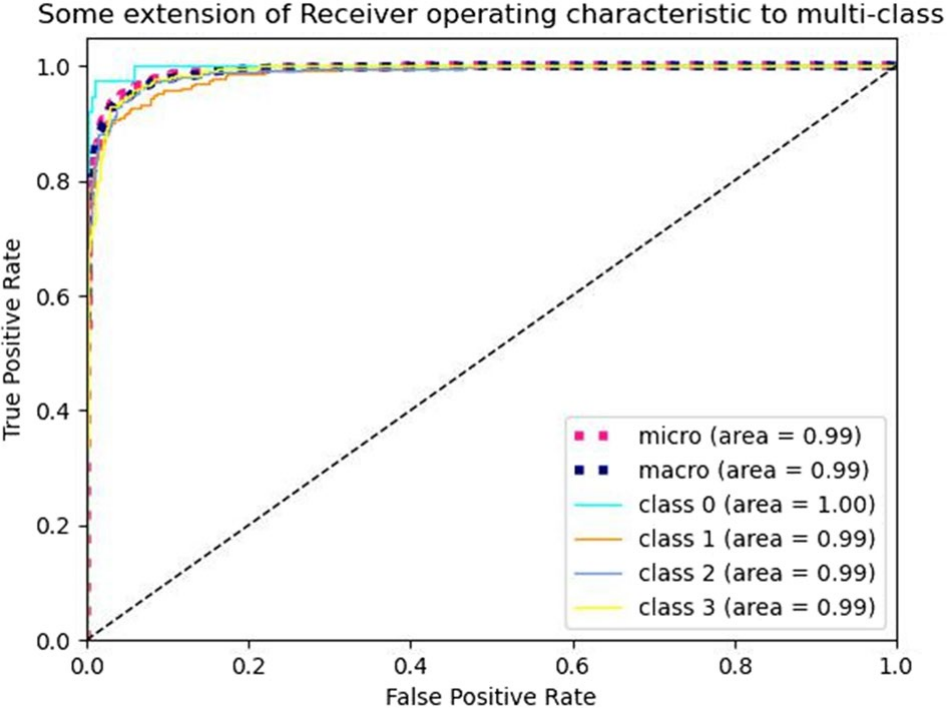
ROC curve of HAHNet

In this experiment, a novel convolutional neural network, HAHNet, is proposed for the multi-stage classification of breast cancer HER2 status. To evaluate the model performance, the accuracy, precision, recall, F-score, and MCC were calculated. Figure 8 presents the ROC curve of HAHNet. The ROC curve is a graphical representation used to describe the performance of a classification model and serves as a comprehensive evaluation metric. Based on the ROC curve, we calculated the AUC (Area Under the Curve) values of HAHNet for predicting 4 levels of HER2 expression. The AUC value reached 0.99, demonstrating that HAHNet performs well in predicting HER2 expression levels. From Fig. 8, it is evident that HAHNet exhibits impressive predictive performance for the 4 HER2 expression levels in the dataset. The calculation formulas of all the evaluation metrics are as follows (Eqs. 3–7). *TP* is a positive sample predicted as a positive class, *TN* is a negative sample predicted as a negative class, *FP* is a negative sample predicted as a positive class, and *FN* is a positive sample predicted as a negative class.

3 $$\begin{aligned}&Accuracy=\frac{TP+TN}{TP+TN+FP+FN} \end{aligned}$$

Accuracy: The proportion of correctly predicted samples to the total samples.

4 $$\begin{aligned}&Recall=\frac{TP}{TP+FN} \end{aligned}$$

Recall: The probability of being predicted as a positive sample in the actual positive sample.

5 $$\begin{aligned}&Precision=\frac{TP}{TP+FP} \end{aligned}$$

Precision: The probability of the actual positive sample among all the samples predicted to be positive.

6 $$\begin{aligned}&F\text {-}score=\frac{2\times TP}{2\times TP+FP+FN} \end{aligned}$$

F-score: The harmonic mean of precision and recall, which is closer to the smaller value of the two numbers.

7 $$\begin{aligned}&M C C=\frac{T P \times T N-F P \times F N}{\sqrt{(T P+F P) \times (T P+F N) \times (F P+T N) \times (F N+T N)}} \end{aligned}$$

MCC: It is essentially a correlation coefficient that describes the actual classification and the predicted classification. The value closer to 1 indicates the better prediction effect.

### Experimental results

**Fig. 9:**
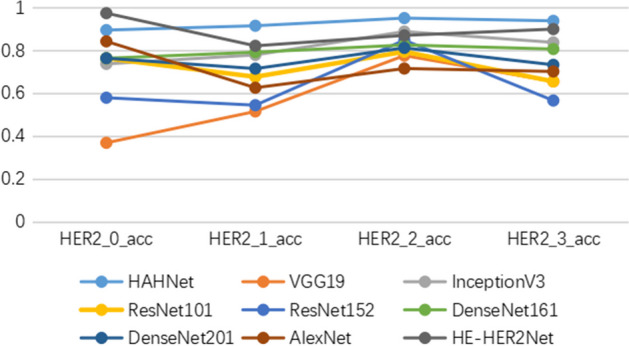
This figure presents a comparison of the performance of nine models participating in the comparative experiment in predicting the four HER2 expression statuses

HAHNet was compared with AlexNet, VGG19, InceptionV3, ResNet101, ResNet152, DenseNet161, Densenet201 and HE-HER2Net. Figure 9 demonstrates the comparisons of prediction effects of all models on the four HER2 statuses. It is seen intuitively that HAHNet performs suboptimally only in the HER2$\_$0 category, while it achieves the best predictive performance in the HER2$\_$1+, HER2$\_$2+, a$\_$d HER2$\_$3+ categories. Overall, HAHNet demonstrates superior overall performance compared to other methods.

**Fig. 10:**
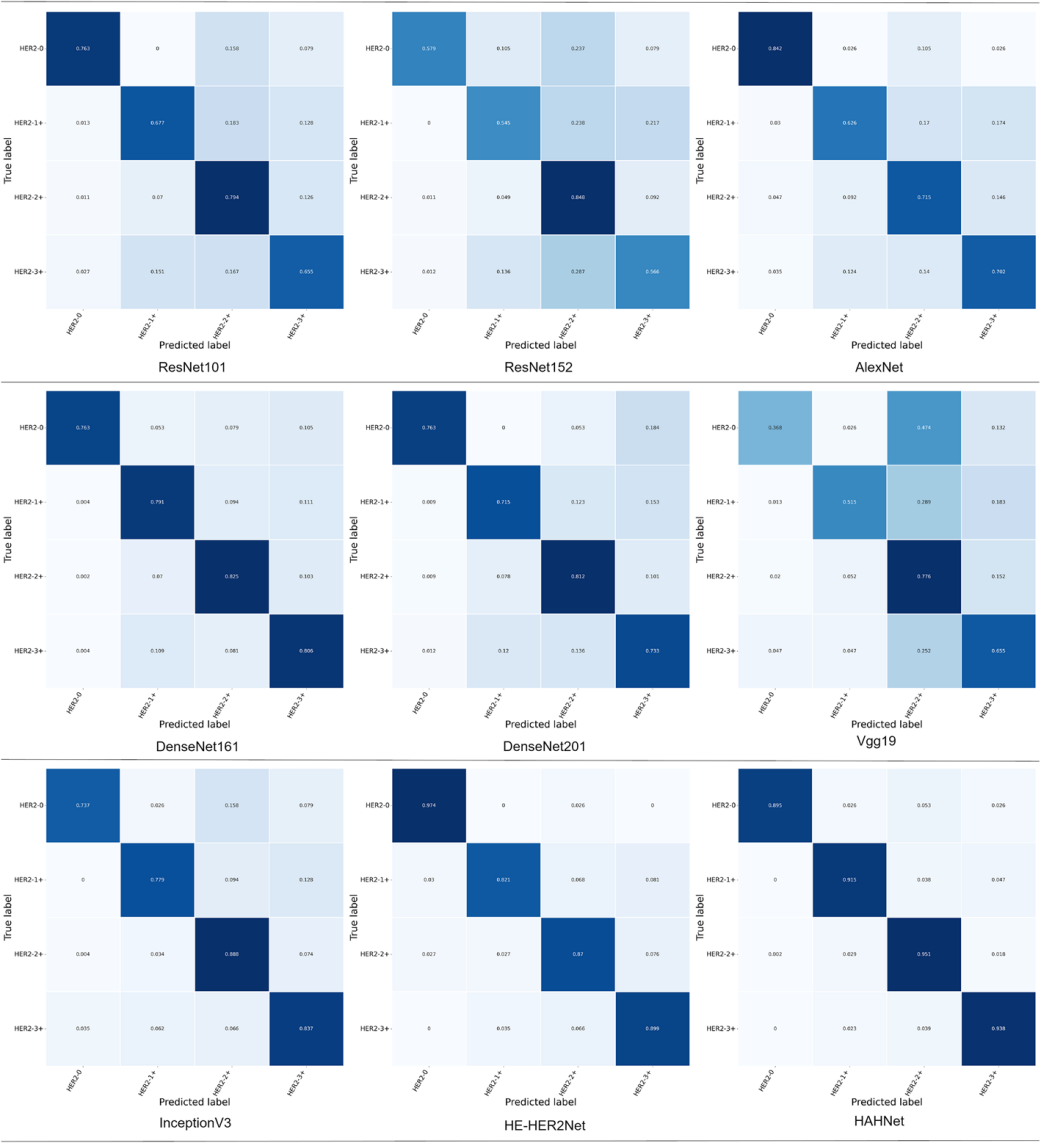
This figure displays the performance of the confusion matrices for the nine models, namely ResNet101, ResNet152, AlexNet, DenseNet161, DenseNet201, Vgg19, InceptionV3, HE-HER2Net, and HAHNet, which participated in the comparative experiment

The confusion matrix of the above models was introduced to further analyze all the models. Figure 10 displays a diagram of the confusion matrix of all models. The dark blue modules on the diagonal in the confusion matrix represented the accuracy of the model for each type of prediction, in HER2-1+, HER2-2+, and HER2-3+ categories. The accuracy rates of HAHNet in the prediction of three categories were 0.915, 0.951, and 0.938, respectively, higher than all the other compared models.

**Fig. 11:**
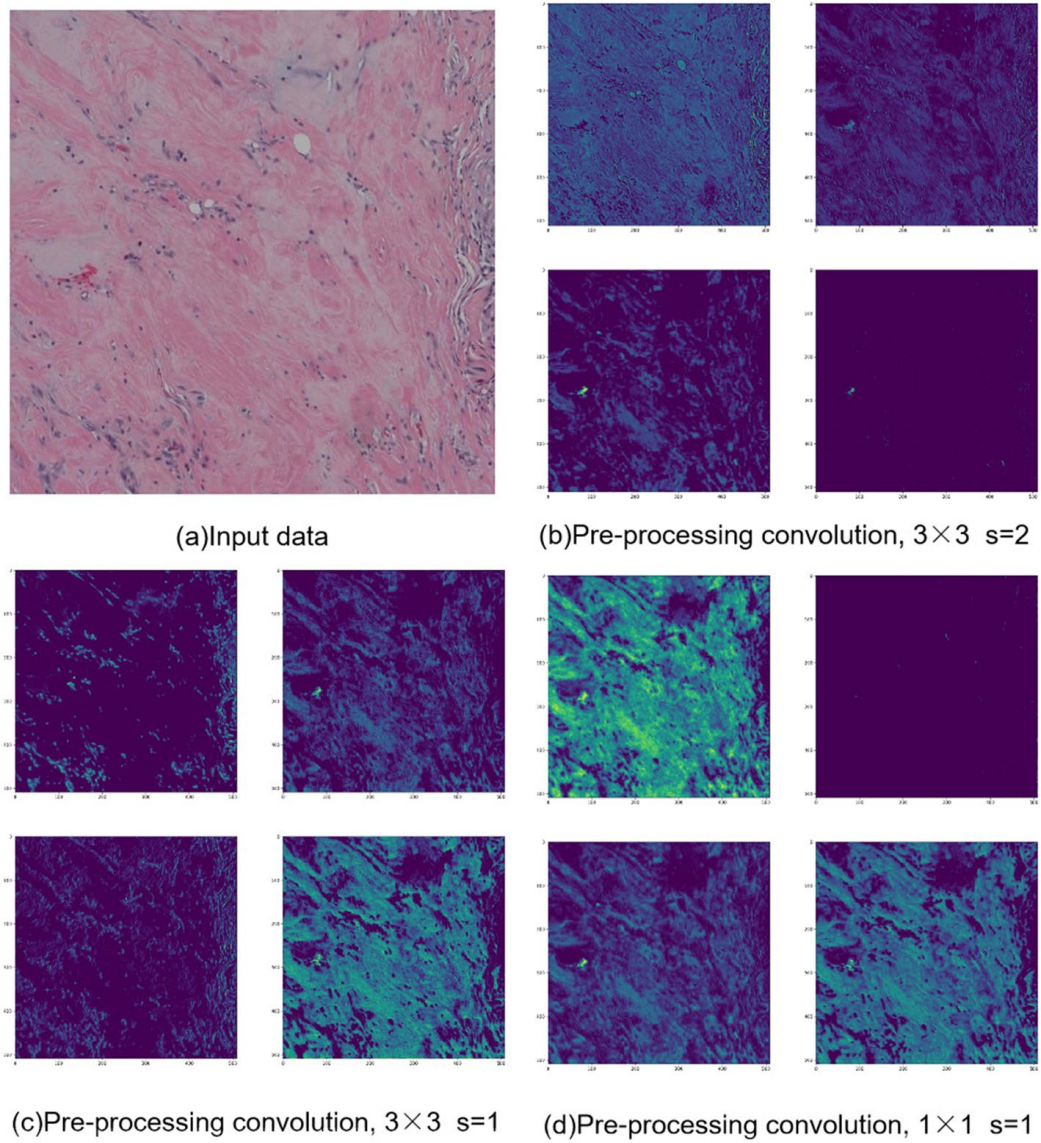
The data that are incorrectly predicted by the serial models such as ResNet101, ResNet152, ResNet161, and ResNet201, but correctly predicted by the parallel models. **a** denotes unprocessed data, **b**, **c**, **d** are feature maps generated by HAHNet convolution. The convolution order starts with the convolution in (**b**), followed by (**c**), and finally (**d**). The convolutions in **b**, **c**, and **d** each consist of a single convolutional layer and are repeated only once. We extract and display the feature maps corresponding to four channels from each convolution. The highlighted part of the feature map is the feature noticed by the model

Figure 11 presents the original image and feature maps of the image that is correctly predicted by the parallel model and incorrectly predicted by the serial model, where the feature maps are obtained by HAHNet processing. Compared with the rest of the data, the image has a lower degree of dyeing and a wider distribution of features, which make it more difficult to extract the image features, and some small features and association information between regions are easily lost during processing.

Table 1 describes the comparisons between HAHNet and other models on multiple metrics. Obviously, HAHNet outperforms all the other compared models. Compared with InceptionV3, HAHNet shows great improvements in the six evaluation metrics of acc, precision, recall, F-score, MCC, and AUC. Relative to the suboptimal method HE-HER2Net, the accuracy rate of HE-HER2Net is 0.8701, and that of HAHNet is 0.9365, which increases by about 0.065. The precision of HE-HER2Net is 0.8773, and that of HAHNet is 0.9367, which elevates by 0.06. Moreover, the recall rate of HE-HER2Net is 0.8700, and that of HAHNet is 0.9246, with an increase of about 0.055. Furthermore, the F-score of HE-HER2Net is 0.8711, and that of HAHNet is 0.9366, showing an increase of approximately 0.065. The MCC of HE-HER2Net is 0.8076, and that of HAHNet is 0.9041, demonstrating an increase of around 0.1.

**Table 1:** This table presents the performance evaluation results of all models in the comparative experiment

Model	Accuracy	Precision	Recall	F-score	MCC	AUC
HAHNet	0.9365	0.9367	0.9246	0.9366	0.9041	0.99
HE-HER2Net	0.8701	0.8773	0.8700	0.8711	0.8076	0.91
InceptionV3	0.8423	0.8448	0.8101	0.8428	0.7629	0.88
DenseNet161	0.8096	0.8156	0.7964	0.8112	0.7159	0.87
DenseNet201	0.7656	0.7686	0.7556	0.7667	0.6484	0.84
ResNet101	0.7277	0.7280	0.7221	0.7276	0.5898	0.82
ResNet152	0.6899	0.6852	0.6342	0.6832	0.5231	0.79
AlexNet	0.6949	0.7089	0.7211	0.6981	0.5524	0.80
Vgg19	0.6653	0.6741	0.5785	0.6621	0.4894	0.72

### Ablation experiment

Ablation experiments are also designed in this study. In each experiment, one block in HAHNet is removed while the rest are kept. The results are shown in Table 2. In HAHNet$\_$1, the DownsampleBlock in HAHNet is removed, so that the model is a serial structure, and the final HAHNet$\_$1 accuracy is 0.8843. The original InceptionB and InceptionD blocks in InceptionV3 do not contain the above-mentioned fourth line. In HAHNet$\_$2, the unmodified InceptionB and InceptionD Blocks are utilized, and the accuracy is 0.9181. HAHNet$\_$3 removes the attention block in HAHNet and achieves an accuracy of 0.9242.After comparison, HAHNet achieves the best results with an accuracy rate of 0.9365. This indicates that adopting a parallel structure, modifying the structure of the InceptionB/InceptionD Block, and introducing attention mechanisms effectively enhance the performance of HAHNet in extracting multi-scale features and contextual information.

**Table 2:** Comparison results of ablation experiments

Model	Accuracy	Precision	Recall	F-score	MCC	AUC
HAHNet	0.9365	0.9367	0.9246	0.9366	0.9041	0.99
HAHNet_1	0.8843	0.8923	0.8821	0.8938	0.8522	0.92
HAHNet_2	0.9181	0.9201	0.9126	0.9202	0.8896	0.96
HAHNet_3	0.9242	0.9281	0.9179	0.9285	0.8962	0.97

## Discussion

This study aims to predict the HER2 status from H&E images. To achieve this aim, several classic models, such as AlexNet, VGG19, InceptionV3, ResNet101, ResNet152, DenseNet161, and Dense net201, were trained. Due to the difficult classification of some data in the Dataset, some classic models cannot effectively complete the multi-classification tasks on H&E images. The accuracy, precision, recall rate, and AUC of some models are very low, along with poor loss convergence, and the final loss value is high.

From Table 1, we can clearly see that the parallel models, HAHNet, HE-HER2Net, and InceptionV3, achieve prediction accuracies of 0.9365, 0.8701, and 0.8423, respectively. On the other hand, among the serial models, DenseNet161 performs the best with an accuracy of only 0.8096. We observe that the parallel structure models outperform the other models. To analyze these results, we provide the feature maps extracted during the convolution process. As observed from Fig. 11, the feature sizes in the histological images vary greatly, and the feature distribution is wide. In this regard, capturing multi-scale features and extracting contextual information become the key factors for HER2 status classification. In the convolution block of the parallel structure model, convolution kernels of different sizes are utilized to extract multi-scale features, which reduce feature loss. However, the serial structure model does not have such ability, leading to the even worse effect of the serial structure model. This gives us the new research ideas of retaining the parallel structure of the convolution block, applying the idea of multi-scale feature extraction in the overall network structure design, and introducing the attention mechanism. Based on the above new research ideas, HAHNet is proposed in this paper. Our results show that the proposed HAHNet is able to classify the HER2 status of H&E images with high accuracy, and our method achieves excellent results on different evaluation metrics.

In general, the novelty of the proposed HAHNet can be summarized as follows: (1) It predicts HER2 expression levels in breast cancer based on H&E images, which greatly reduces the cost of HER2 expression level prediction. (2) HAHNet adopts a parallel structure and designs two parallel feature extraction lines. The two lines are responsible for feature extraction on images of different sizes to obtain more multi-scale features, so that the model has much more choices. (3) The parallel structure DownsampleBlock is adopted during the downsampling process to better collect detail features, organizational structural features, and image context information during the image downsampling process. (4) The improved InceptionB and InceptionD modules are able to extract global spatial information in the dimensionality reduction operation, which is very important for the final prediction results. Moreover, the introduction of more activation functions in the module effectively improves the model learning ability. (5) The introduced attention mechanism assigns greater weights to important features, thus properly directing the attention of the model to ignore irrelevant information, amplify important information, and guide the model to learn important features. This paper introduces a new attention mechanism called Eca-CBAM attention mechanism. The Eca-CBAM attention mechanism improves upon the CBAM attention mechanism by avoiding the loss caused by dimension reduction operations.

Breast cancer is a significant health issue among women, and HER2 is a crucial prognostic and predictive factor. The classification of HER2 status is essential for determining treatment plans for breast cancer. Our method employs H&E images to discriminate HER2 status. Moreover, based on the experimental results presented in this paper, HAHNet demonstrates significant improvements over HE-HER2Net, another HER2 expression prediction method using H&E images. Specifically, HAHNet achieves approximately 6.5% higher accuracy, 6% higher precision, 5.5% higher recall, 6.5% higher F1-score, and 10% higher MCC compared to HE-HER2Net. HAHNet effectively addresses the issues of high cost and inadequate accuracy in the current field of HER2 status classification. It can serve as a valuable reference for pathologists in HER2 breast cancer screening, diagnosis, and prognosis decision-making.

## Conclusion

In conclusion, a parallel-structured neural network is presented in this paper. The idea of multi-scale feature extraction is applied in the convolutional module and network structure design, which combines with attention mechanisms, effectively improves the accuracy of HER2 status classification on H&E stained images. The results show that the proposed HAHNet is efficient in HER2 status evaluation on H&E stained images. For the HER2 classification task, HAHNet achieves the accuracy of 0.9365, precision of 0.9367, recall of 0.9246, F-score of 0.9041, and AUC of 0.99, demonstrating higher performance than the existing methods. With regard to the analysis of feature maps and model structure, the reasons for the high efficiency of HAHNet are explained, which makes our model more transparent.

Breast cancer histological images are a type of complex structured data. Although HAHNet utilizes a multi-scale feature fusion approach to extract features from different scales, deep learning methods primarily focus on feature representation and learning, making them more suitable for uncovering local patterns in the data. This means that some global structural features in the images may be overlooked. Additionally, deep learning methods may encounter challenges in computation and storage when dealing with large-scale data. In contrast, graph theory methods emphasize the topological structure and connectivity between nodes, which is crucial for studying the global properties of data. Some graph theory methods have already emerged in the medical field. Rostami et al. [29] introduced the application of community detection algorithms in the healthcare domain, and Azadifar et al. [30] proposed a graph-based gene selection method for cancer diagnosis. It is important to emphasize that graph theory methods and deep learning methods are not mutually exclusive but can be combined and integrated. When dealing with complex data and tasks, combining graph theory methods with deep learning can leverage the strengths of both approaches to improve model performance and representation. Therefore, integrating graph theory methods into deep learning models may lead to better predictions of HER2 status in breast cancer.

In future work, we aim to develop a model that combines image synthesis capabilities with HER2 expression prediction. This model will generate IHC-stained images based on H&E images and utilize the generated IHC-stained images to further predict the HER2 expression levels within the images. During the prediction process, we will incorporate some graph theory related algorithms. By doing so, we expect to achieve improved predictive performance and provide greater assistance to pathologists.

## Data Availability

The data used in this work comes from https://bupt-ai-cz.github.io/BCI/.
